# Cerebral White Matter Lesions and Affective Episodes Correlate in Male Individuals with Bipolar Disorder

**DOI:** 10.1371/journal.pone.0135313

**Published:** 2015-08-07

**Authors:** Armin Birner, Stephan Seiler, Nina Lackner, Susanne A. Bengesser, Robert Queissner, Frederike T. Fellendorf, Martina Platzer, Stefan Ropele, Christian Enzinger, Petra Schwingenschuh, Harald Mangge, Lukas Pirpamer, Hannes Deutschmann, Roger S. McIntyre, Hans-Peter Kapfhammer, Bernd Reininghaus, Eva Z. Reininghaus

**Affiliations:** 1 Department of Psychiatry, Medical University of Graz, Graz, Austria; 2 Department of Neurology, Medical University of Graz, Graz, Austria; 3 Division of Neuroradiology, Department of Radiology, Medical University of Graz, Graz, Austria; 4 Research Unit on Lifestyle and Inflammation-associated Risk Biomarkers, Clinical Institute of Medical and Chemical Laboratory Diagnostics, Medical University Graz, Graz, Austria; 5 Mood Disorders Psychopharmacology Unit at the University Health Network, University of Toronto, Toronto, Canada; University of Oxford, UNITED KINGDOM

## Abstract

**Background:**

Cerebral white matter lesions (WML) have been found in normal aging, vascular disease and several neuropsychiatric conditions. Correlations of WML with clinical parameters in BD have been described, but not with the number of affective episodes, illness duration, age of onset and Body Mass Index in a well characterized group of euthymic bipolar adults. Herein, we aimed to evaluate the associations between bipolar course of illness parameters and WML measured with volumetric analysis.

**Methods:**

In a cross-sectional study 100 euthymic individuals with BD as well as 54 healthy controls (HC) were enrolled to undergo brain magnetic resonance imaging using 3T including a FLAIR sequence for volumetric assessment of WML-load using FSL-software. Additionally, clinical characteristics and psychometric measures including Structured Clinical Interview according to DSM-IV, Hamilton-Depression, Young Mania Rating Scale and Beck’s Depression Inventory were evaluated.

**Results:**

Individuals with BD had significantly more (*F* = 3.968, *p* < .05) WML (Mdn = 3710mm3; IQR = 2961mm3) than HC (Mdn = 2185mm3; IQR = 1665mm3). BD men (Mdn = 4095mm3; IQR = 3295mm3) and BD women (Mdn = 3032mm3; IQR = 2816mm3) did not significantly differ as to the WML-load or the number and type of risk factors for WML. However, in men only, the number of manic/hypomanic episodes (*r* = 0.72; *p <* .001) as well as depressive episodes (*r* = 0.51; *p* < .001) correlated positively with WML-load.

**Conclusions:**

WML-load strongly correlated with the number of manic episodes in male BD patients, suggesting that men might be more vulnerable to mania in the context of cerebral white matter changes.

## Introduction

The neurobiological basis of bipolar disorder (BD) is not sufficiently characterized. Structural brain abnormalities in BD are reported as a common feature in neuroimaging studies. For example, white matter lesions (WML, also termed white matter hyperintensities) are hyperintense bright spots that are detectable at T2-weighted and fluid-attenuated inversion recovery (FLAIR) sequences on Magnetic Resonance Imaging (MRI) of the brain and are one of the most consistently reported brain abnormalities in individuals with BD [1, 2]. White matter lesions have conventionally been classified by location into those that occur in the periventricular or deep white matter [3]. Individuals with BD are approximately 2.5 times more likely to have WML compared to controls, with deep WML differentially represented [1, 2].

White matter lesions are not specific to BD, as they are also found in normal aging and are more common in individuals with cardio- and cerebrovascular disease as well as in various neuropsychiatric conditions (e.g. as unipolar depression, schizophrenia, panic disorder, substance abuse/dependency, migraine and some forms of dementia) [4–12]. Two studies reported associations between WML and alcohol dependency [13, 14], while a separate study which investigated WML and alcohol intake controlling for potential confounders did not show any associations [15].

There are several possible non-mutually exclusive causes of WML, including ischemia, demyelination, edema, and gliosis. Early confluent and confluent WML have been shown to more typically represent ischemic tissue damage [3] and progress over time [16]. In otherwise healthy adults, the presence of WML is generally associated with age and cardiovascular risk factors such as hypertension or smoking [17, 18]

Adults with BD are at increased risk of cardiovascular disease and hypertension compared to non-BD adults [19]. Additionally, smoking behavior and obesity/metabolic syndrome differentially affect individuals with BD [20, 21]. Moreover, replicated evidence implicates that the occurrence of obesity and/or the metabolic syndrome is associated with a more complex BD illness presentation and course [21–26], worse cognitive function [27] and increased rate of cerebro- and cardiovascular disease in BD [21]. Available evidence suggests that cognitive dysfunction in BD may be related to WML in some circumstances [28].

White matter lesions are also predictors for depressive symptoms [17]. Confluent severe deep WML may transform into true infarcts [18]. Infarcts are in approximately 80% of ischemic origin [29] and are capable of triggering depression and in rare cases also mania. A further line of evidence is the observation that up to thirty percent of individuals with post-stroke mania might also develop BD [30–32].

Several associations between clinical characteristics and WML in BD have been described in the literature. Positive correlations with the number of hospitalizations and suicide attempts, and poor illness outcome as well as poorer treatment response, decreased performance on neuropsychological tests and impaired insight have been reported [7, 33–38].

Investigations of WML in BD have relied on a categorical method of lesion description reporting the presence or absence of white matter lesions and grading this presence on scales of differing reliabilities and measurement characteristics [39–41].

Although volumetric analysis is common within the field of structural neuroimaging, this approach has rarely been applied for rating WML in BD [38, 42, 43].

The foregoing collection of observations provided the impetus for exploring the following hypotheses in euthymic adults with BD: (I) BD individuals exhibit a higher WML-load than healthy controls. And, (II) there are associations between clinical characteristics and WML-load in BD. A particular emphasis was given on concurrent obesity / metabolic syndrome and the moderational influence of sex.

## Methods and Materials

The study was conducted at the Medical University of Graz, Department of Psychiatry. All patients took part in the ongoing single center BIPFAT study, that assesses demographic parameters, complete actual and lifetime psychiatric history using the Structured Clinical Interview according to DSM-IV (SCID I), the psychiatric rating scales Hamilton-Depression (HAM-D) [44], Young Mania Rating Scale (YMRS) [45] and Beck’s Depression Inventory (BDI) [46], history of medication, anthropometric measure, blood pressure, fasting blood, cognitive testing, EEG, stool sample, different lifestyle questionnaires and magnetic resonance imaging (MRI) of the brain. All patients included were former in- or outpatients of the Medical University of Graz and had a diagnosis of BD I or BD II according to the DSM-IV criteria. Patients needed to be in the state of euthymia (HAM-D score <11 and YMRS <9) and had given written informed consent prior to participating in the study.

Exclusion criteria were the presence of chronic obstructive pulmonary disease, rheumatoid arthritis, systemic lupus erythematosus, inflammatory bowel disease, neurodegenerative and neuroinflammatory disorders (i.e. Alzheimer's, Huntington's and Parkinson's disorder, multiple sclerosis), hemodialysis and interferon-α-based immunotherapy. Further exclusion criteria for controls were the presence of lifetime psychiatric diagnoses (verified by SCID I) and first and second grade relationship to relatives with psychiatric disorders. For further information about the study design and preliminary results see our previous reports [47–49]

One hundred BD patients (52 male, 48 female) and 54 healthy controls (HC, 23 male, 31 female) have been included in this study.

### Ethics statement

The study has been approved by the local ethics committee (Medical University of Graz, Austria) in compliance with the current revision of the Declaration of Helsinki, ICG guideline for Good Clinical Practice and current regulations (EK-number: 24–123 ex 11/12).

### Magnetic resonance imaging

MRI was performed on a 3T whole body scanner (TimTrio; Siemens Healthcare, Erlangen, Germany).

The protocol included an axial FLAIR sequence (TR = 10000ms, TE = 69ms, inversion time = 2500ms, number of slices = 40, slice thickness = 3mm, in-plane resolution = 0.86x0.86 mm²) and a high resolution T1 weighted 3D sequence with magnetization prepared rapid gradient echo (MPRAGE) and whole brain coverage (TR = 1900ms, TE = 2.19ms, inversion time = 900ms, flip angle = 9°, isotropic resolution of 1 mm).

Supra- and infratentorially located lesions of high signal intensity on FLAIR images were considered as WML. Silent non-lacunar infarcts and lacunes were excluded from the analysis. Non-lacunar infarcts were lesions with typical signal characteristics of infarcts following a typical vascular territory or being located in a border zone between two vascular territories. Lacunes were focal lesions involving the basal ganglia, the internal capsule, the thalamus, the brainstem or the white matter not exceeding a maximum diameter of 20 mm [50, 51].

**Fig 1:**
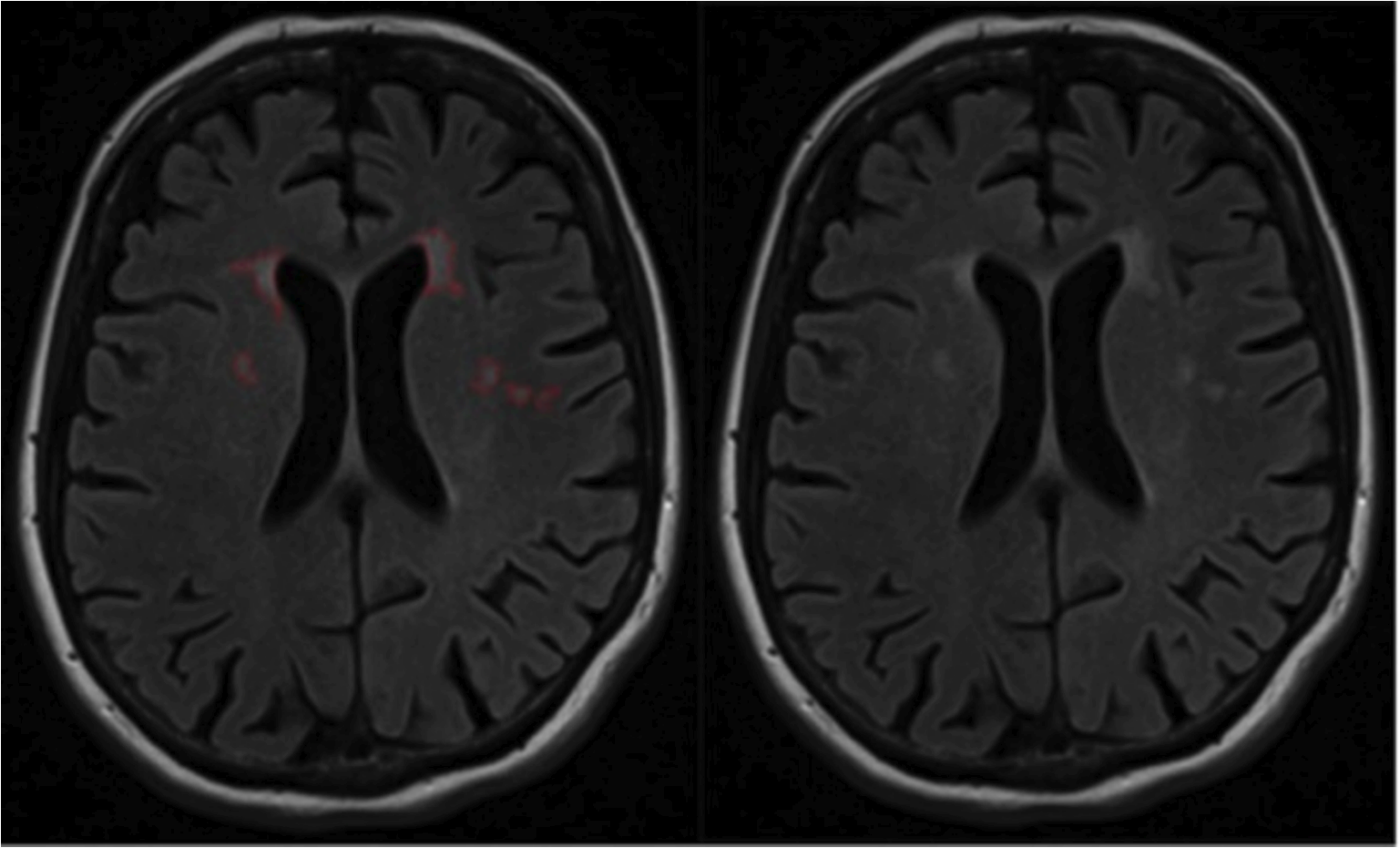
Representative FLAIR slice of one patient. The left image shows an example of lesion areas, outlined semiautomatically by the instructed rater using a custom written IDL program (Exelis Visual Information Solutions, USA). The right image shows the native FLAIR scan.

WML were outlined on a computer using a custom written IDL program (Exelis Visual Information Solutions, USA; see Fig 1). Lesion areas were segmented by combined region growing and local thresholding following manual selection by a single instructed rater [52]. To counteract potential inter-rater variability, 15 cases from the study cohort were randomly chosen and re-rated by the instructor, a neurologist who is highly experienced in identification, volumetry and rating of WML. The neurologist was blinded to previous results and outlined WML on FLAIR scans of the 15 cases using the same procedure as the main rater. WML volumes of both raters were entered into SPSS. Subsequent intraclass correlation analysis yielded an intraclass correlation coefficient (ICC (2, 1)) of 0.938 (95%CI 0.570–0.984; p < .001). An ICC of 0.938 indicates high agreement and supports the reliability of WML volumetry between the two raters. Lesion volume in mm3 was calculated using the program FSLMATHS (FSL, Oxford, www.fmrib.ox.ac.uk) by multiplying the lesion area with the slice thickness and normalized by total intracranial volume (TIV). Segmentation of TIV and cortex volume from the T1-weighted high resolution MPRAGE scans was performed with the Freesurfer image analysis suite, which is documented and freely available for download online (http://surfer.nmr.mgh.harvard.edu/) [53, 54].

We decided against a separation into deep and periventricular WML as the drawing of rather random borders in the lesions themselves was often considered to be arbitrary, which was especially the case in large periventricular lesions conflating into the deep white matter.

### Statistics

Student´s *t*-tests were performed for group comparisons of normally distributed variables. Categorical data sets were analyzed using chi-squared tests. Tests were two tailed and a value of *p* < .05 was considered statistically significant. For group differences normal distribution was confirmed using Kolmogorov Smirnov test. In case of violation of normality, Mann-Whitney-U-tests were performed. For differences in WML-load, BD patients and controls were analyzed using a univariate covariance analysis model (ANCOVA) controlling for the confounding factor age and additionally for BMI, smoking, and diabetes, as the groups differed in these parameters.

For partial correlation analyses, variables of interest matching the required normal distribution of residuals confirmed visually by histograms have been used for calculation (normalized WML with number of depressive episodes, number of manic episodes, illness duration, age of onset and BMI). For this purpose normalized WML volumes have been transformed by adding the number one and the use of a natural logarithm. There was no normal distribution of residuals for the rating scale variables YMRS, HAM-D and BDI forcing us to remove them from partial correlation analysis. After Bonferroni-correction for multiple comparison a value of *p* < .01 was considered statistically significant. Partial correlation analyses of WML were conducted controlling for age, hypertension, diabetes, smoking, migraine, alcohol dependency, substance dependency, anxiety disorder and medication (lithium, antiepilieptics and antipsychotics). Illness duration was additionally introduced as a control variable for number of affective episodes and age of onset.

## Results

### Differences BD and HC

Group differences between BD patients and controls can be seen in Table 1. ANCOVA results indicated that individuals with BD had significantly more WMLs than HC (*F(1/148)* = 3.968, *p* = .048, *Eta* = .026; Median and IQR are shown in Table 1).

**Table 1:** Demographic data.

	BD (n = 100)	HC (n = 54)	*Statistics*	*p*
**Male (%)**	52	42.6	*χ 2 = 1*.*242*	.*265*
**Age (years) (M, SD)**	44 (14)	41 (16)	*U = -1*.*475*	.*140*
**Body Mass Index (M, SD)**	28 (5)	25 (4)	***t = 3*.*695***	.*000*
**Migraine (%)**	22	27.8	*χ 2* = .*750*	.*386*
**Hypertension (%)**	29	22.2	*χ 2* = .*825*	.*364*
**Smoking (%)**	49	24.1	***χ 2 = 9*.*058***	.*003*
**Diabetes (%)**	7	0	***χ 2 = 3*.*960***	.*047*
**WML (mm³) (Mdn, IQR)** ^1^	3710 (2961)	2185 (1665)	***F = 3*.*968***	.*048*

Note: Results from *t*-tests (*t*), Chi square tests (*χ2*), Mann-Whitney-U-tests (*U*) and ANCOVA (*F*)

(1) For ANCOVA, WML volumes were normalized by individual total intracranial volume and controlled for age, diabetes, smoking and BMI.

Statistically significant effects are marked bold.

### Sex Differences within BD

Descriptive baseline characteristics within the BD group (males vs. females) are displayed in Table 2. In BD patients, there was no significant sex difference for demographic, clinical or vascular risk parameters.

**Table 2:** Clinical characteristics in BD stratified by sex.

	52 males	48 females	*Statistics*	*p*
**BD I / BD II (%)**	65.4 / 34.6	64.6 / 35.4	*χ 2* = .*007*	.*933*
**Age (years) (M, SD)**	44 (14)	44 (14)	*t = -0*.*24*	.*981*
**Illness Duration (years) (M, SD)**	19 (13)	20 (19)	*U = -*.*235*	.*814*
**Age First Episode (years) (M, SD)**	25 (12)	25 (9)	*U = -*.*366*	.*714*
**Manic/hypomanic Episodes (M, SD)**	11 (18)	8 (9)	*U = -*.*603*	.*547*
**Depressive Episodes (M, SD)**	12 (12)	16 (15)	*U = -*.*513*	.*608*
**History of Suicide Attempts (%)**	38.5%	27.1	*χ 2 = 1*.*462*	.*227*
**HAM-D (M, SD)**	5 (4)	6 (5)	*U = -*.*573*	.*567*
**Alcohol Dependency (%)**	23.1	12.5	*χ 2 = 1*.*892*	.*169*
**Substance Dependency (%)**	11.5	10.4	*χ 2* = .*032*	.*858*
**Anxiety Disorder (%)**	17.3	29.2	*χ 2 = 1*.*840*	.*175*
**YMRS (M, SD)**	1 (2)	1 (2)	*U = -1*.*665*	.*095*
**BDI (M, SD)**	12 (9)	15 (12)	*U = -1*.*254*	.*210*
**Body Mass Index (M, SD)**	28 (4)	28 (6)	*t = -0*.*56*	.*955*
**Antidepressant (%)**	44.2	31.3	*χ 2 = 1*.*785*	.*182*
**Lithium (%)**	23.1	10.4	*χ 2 = 2*.*835*	.*092*
**Antiepileptic (%)**	25	12.5	*χ 2 = 2*.*534*	.*111*
**Atypical Antipsychotic (%)**	42.3	39.6	*χ 2* = .*077*	.*782*
**Typical Antipsychotic (%)**	11.5	14.6	*χ 2* = .*205*	.*651*
**Migraine (%)**	23.1	20.8	*χ 2* = .*073*	.*787*
**Hypertension (%)**	30.7	27.1	*χ 2* = .*165*	.*685*
**Smokers (%)**	48.1	50	*χ 2* = .*037*	.*848*
**Diabetes (%)**	7.7	6.3	*χ* ^*2*^ = .*080*	.*778*
**WML (mm³) (*Mdn*, IQR)** ^1^	4095 (3295)	3032 (2816)	*U = -*.*150*	.*133*

Note: Results from *t*-tests (*t*), Chi square tests (*χ* ^2^) and Mann-Whitney-U-tests (*U*).

(1) WML were normalised by individual total intracranial volume for the calculations.

### Correlations of WML with clinical and demographic factors

The results of partial correlation analyses of WML with clinical and demographic characteristics within the BD group are presented in Table 3. WML-load correlated positively with manic episodes and illness duration. In BD men, the number of manic / hypomanic episodes as well as depressive episodes correlated positively with WML-load. In BD women, no significant correlations were found.

**Table 3:** Partial correlation analyses.

	WML-load		
	*BD (n = 100)*	*BD male (n = 52)*	*BD female (n = 48)*
**Manic episodes** ^1^	**.405** ***	**.723** ***	-.075
**Depressive episodes** ^1^	.220*	**.510** ***	.067
**Age of onset** ^1^	-.158	.093	-.316
**Body Mass Index** ^2^	-.020	-.119	.104
**Illness duration** ^2^	**.295** **	.383*	.068

Note: WML have been normalized by total intracranial volume and have been transformed by adding the number one and the use of natural logarithm

(1) controlled for age, hypertension, diabetes, smoking, alcohol dependency, substance dependency, anxiety disorder, migraine, lithium, antiepileptics, antipsychotics and illness duration

(2) controlled for age, hypertension, diabetes, smoking, migraine, alcohol dependency, substance dependency, anxiety disorder, lithium, antiepileptics and antipsychotics; Significant results after Bonferroni correction for multiple comparison (*p* < .01) are shown in bold

*p < .05

*p* < .01**

*p* < .001***.

## Discussion

There are several important observations that emerge from the analysis of our data. First, we found an increased total volume of WML in individuals with BD compared to HC. Secondly, the most striking result was the strong association between the number of manic and depressive episodes with the total volume of WML in men. Thirdly, illness duration was identified as a significant and independent factor associated with the volume of WML. In contrast we identified no significant correlation between BMI or age of onset and WML-load in BD.

Our results of increased WML in BD compared to HC can be understood in the context of existing literature [1,2]. We explain the higher occurrence of WML in BD by the fact that WML are simply more common in various neuropsychiatric disorders (BD being one of them) as well as in cardiovascular diseases (more likely to appear in individuals with BD). The underlying pathophysiology might involve processes of inflammation [22], oxidative stress [49], metabolic neurotoxicity [47] and vascular genesis [19–21].

Men and women in our cohort did neither differ significantly in the total volumes of WML nor in the number of affective episodes. Nevertheless the correlation between the number of affective episodes and WML was only present in men. The sex effect found in our study may be a consequence of the neuroprotective influence of estradiol and progesterone (e.g. decrease of oxidative stress) [55–58]. Estrogen and progesterone may influence DNA repair, activation of antioxidative defense and interaction with BDNF [58–61]. Furthermore, decreased estrogen levels have been associated with poor cognitive performance, especially in short term verbal memory function in women [62, 63]. Bae et al. [11] displayed a higher amount of WML in male methamphetamine abusers than in female methamphetamine abusers. They assumed that estrogen's protective effect against cerebrovascular accidents might have been responsible for this result.

Neither do we know if WML in men are responsible for a poorer illness outcome nor if WML subserve clinical symptoms of manic and/or depressive episodes. In this context it is interesting that infarcts are capable to trigger depression and in rare cases also mania. Severe WML are proposed to be of ischemic origin, and may transform into true infarcts [18]. Interestingly, a systematic review on stroke and mania involving 74 cases showed that men are approximately three times more likely to express post-stroke mania than women [64]. Stroke in general is more prevalent in men [65, 66]. However, this cannot explain the much higher occurrence of post-stroke mania in male individuals.

As a result of the foregoing observations we propose that the male brain may be more differentially vulnerable to manic affective symptoms in the context of white matter changes. On the other hand, depression after stroke is not more common in men than in women [67]. We assume that the positive correlation with depressive episodes in our study is a consequence of the direct correlation with manic episodes.

In general, sex differences in the course of BD have been repeated. In the study of Kawa et al. [68] more men than women reported mania as first illness episode at the onset of BD I. Rapid cycling might be slightly more common in women, while age at onset and number of affective episodes of each polarity did not differ between men and women in previous studies [68–70]. Men had higher rates of comorbid substance abuse and gambling, while women reported higher rates of comorbid eating disorders, weight change, appetite change and middle insomnia during depression [68]. Interestingly, we could show that high frequencies of weight change, also called weight cycling, were independently related to the number of affective episodes in women with BD only [48].

BMI did not show a significant association with WML in the present study. BMI can be regarded as a proxy of metabolic dysfunction but is not deterministic of metabolic dysfunction. Metabolic obesity is associated with white matter changes in non-psychiatric samples [71–73]. The non-association between BMI and WMLs in our sample could be because no association exists, or because the association between BMI and WML in bipolar adults may only occur in individuals with elevated BMI and associated metabolic morbidity (e.g. dyslipidemia, hypertension etc.), which has to be explored in large samples.

### Limitations

We do not know the causal relationship between WML and clinical symptoms as only correlations were studied. It is not clear whether these lesions are a result of comorbid conditions, or whether they are directly associated with the disorder, or represent a biological risk factor for BD. Age is the most important factor contributing to WML [18] which suggests that WML in young subjects might not reflect a reliable indicator as the underlying pathology might not have had enough time to develop from a micro- to a macro-structural level. In line with this, there are conflicting results concerning the presence of WML in children and adolescents with BD. Some found increased WML already in young ages [74–77] while others found BD not associated with increased rate of WMLs in young subjects without comorbid conditions [78]. Non-conventional more advanced MRI methods as DTI might have provided more detailed insights.

With volumetric analysis it is not possible to distinguish large periventricular from confluent deep WML, which puts it on a disadvantage to the more commonly used rating scales [39].

The foregoing study targets an overall assessment of the burden of white matter lesions in the brain. Consequently, conclusions about pathophysiological mechanisms are highly limited, as especially in psychiatric disorders, the precise anatomic localization of white matter lesions might be pivotal for the pathophysiological consequences [1].

The present study is also limited by its natural cross-sectional design.

Advantages of this study were the acquisition of data in a single center, the selection of all lesions with the same criteria by a single rater and the performance of the identical protocol on the same MRI scanner, as well as the volumetric quantitative lesion segmentation allowing refined statistical analyses.

## Conclusions

Our results underline the increased WML-load in individuals with BD and their association with clinical illness variables. Importantly, a sex specific effect was revealed, as the number of affective episodes correlated positively with WML-load in male bipolar study participants only. The moderational influence of sex suggests a possible contribution role of endocrinological and/or pathogenic influences that affect men and women with BD differently. We propose that men are more vulnerable to mania in the context of ischaemic brain alterations as confluent sub-types of WML or stroke, which should stimulate further research into this area, preferably with longitudinal design.
